# Neurocysticercosis Presenting With Status Epilepticus in a Young South African Male Patient in Saudi Arabia: A Case Report and Review of the Literature

**DOI:** 10.7759/cureus.108916

**Published:** 2026-05-15

**Authors:** Syed Nazeer, Zein B Sheikh, Aram F AlHarbi, Razan S Samman

**Affiliations:** 1 Internal Medicine: Infection Control, Prince Mohammed bin Abdulaziz Hospital, National Guard, Madinah, SAU; 2 Medicine, Prince Mohammed Bin Abdulaziz Hospital, National Guard, Madinah, SAU; 3 Internal Medicine, Prince Mohammed bin Abdulaziz Hospital, National Guard, Madinah, SAU

**Keywords:** calcification, infestation, neurocysticercosis, seizure, taenia solium

## Abstract

Neurocysticercosis (NCC), a parasitic infection caused by the larval stage (*Cysticercus cellulosae*) of *Taenia solium*, is a leading cause of seizures in developing countries. Neurocysticercosis is considered rare in the Arabian Peninsula because of the Islamic prohibition on pork ingestion. We report a case of a 34-year-old male patient who had recently immigrated from South Africa to Saudi Arabia and presented to our emergency department with generalized tonic-clonic seizures. Computed tomography of the head revealed multiple intraparenchymal cystic lesions with a central scolex that were surrounded by minimal edema, along with multiple nodular calcifications; serology was positive for cysticercus IgG antibodies. The patient was treated with albendazole followed by praziquantel in conjunction with dexamethasone and achieved optimal recovery after treatment.Although rare in Saudi Arabia, the prevalence of neurocysticercosis is increasing and must be considered in the differential diagnosis of new-onset seizures, especially among immigrants.

## Introduction

Neurocysticercosis (NCC) is a parasitic infection caused by larval-stage *Cysticercus cellulosae* of the tapeworm *Taenia solium (T. solium)* that is recognized as a leading cause of central nervous system (CNS) infections, particularly in developing countries. It is one of the primary risk factors for new-onset seizure activity in those regions, accounting for approximately 30% of epilepsy cases in Sub-Saharan Africa [1,2]. Sub-Saharan Africa, followed by Latin America and Southeast Asia, are recognized as an endemic region for NCC [3].

*T. solium* is transmitted via the fecal-oral route through the ingestion of its eggs, which are often found in undercooked pork, contaminated vegetables, and contaminated water [1,4]. Once *T. solium* is transmitted, the infection course consists of two main stages known as taeniasis and cysticercosis. Taeniasis is a stage at which *T. solium *infects the intestinal tract after consuming raw or undercooked pork. At this stage, infected individuals will shed eggs of the parasite, which are capable of infecting pigs and humans through food contaminated with feces. The subsequent stage, termed cysticercosis, develops following parasitic invasion of internal organs and larval formation into cysts residing at different sites of the human body such as the skin, muscle, eyes, and CNS. When lodged in the CNS, the term neurocysticercosis is applied. The explanation behind cystic organ disposition remains unclear [5]. The clinical course of neurocysticercosis can involve a variety of neurological symptoms, which are thought to depend on the larval stage of the parasite. Common symptoms include headaches, seizures, and signs of increased intracranial pressure [1]. The mechanisms behind seizure induction in neurocysticercosis are not fully understood; however, emerging studies suggest that inflammation and degeneration of brain tissue during the later stages of T. solium infection are associated with seizure activity [2,4].

There have been very few cases of NCC reported in Saudi Arabia over the past decade; most of which are expats or immigrants coming from endemic areas in their origin. Our aim in this report is to highlight the importance of considering NCC when approaching a seizure patient, especially a foreigner in Saudi Arabia. Although more reporting is required, non-endemic areas, such as Saudi Arabia, might have a growing prevalence of NCC given the ongoing increase in expat population.

## Case presentation

A 34-year-old South African male, with no past medical history, who had recently immigrated to Saudi Arabia 6 months earlier, was brought to the emergency department (ED) with a new-onset seizure. A few hours before the ED arrival, he was complaining of a frontotemporal headache, and later, he developed a witnessed generalized tonic-clonic seizure (GTCS) that subsided within two minutes spontaneously. He was then taken to the ED, without regaining his full consciousness, and during assessment, he developed a second attack of GTCS that lasted for three minutes and was aborted by a 3 milligram intravenous midazolam injection. As a result of sustained unconsciousness with a Glasgow Coma Scale (GCS) of 8/15 (Eyes 2, Verbal 1, Motor 5), he underwent emergent intubation to protect his airway for two days.

On physical examination, the patient was hemodynamically stable, had one documented spike of low-grade fever (37.6 °C), no signs of meningism, and no focal neurological deficits were observed. Additionally, no notable findings were observed on examination of the respiratory, cardiovascular, gastrointestinal, and musculoskeletal systems.

Laboratory tests revealed normal whole blood counts and C-reactive protein levels. Blood chemistry revealed an acute kidney injury stage one with a creatinine level of 114 µmol/L (Reference 64-110 µmol/L), compared to the previous baseline of 70 µmol/L. Liver function tests and urine toxicology screening results were unremarkable.

**Figure 1:**
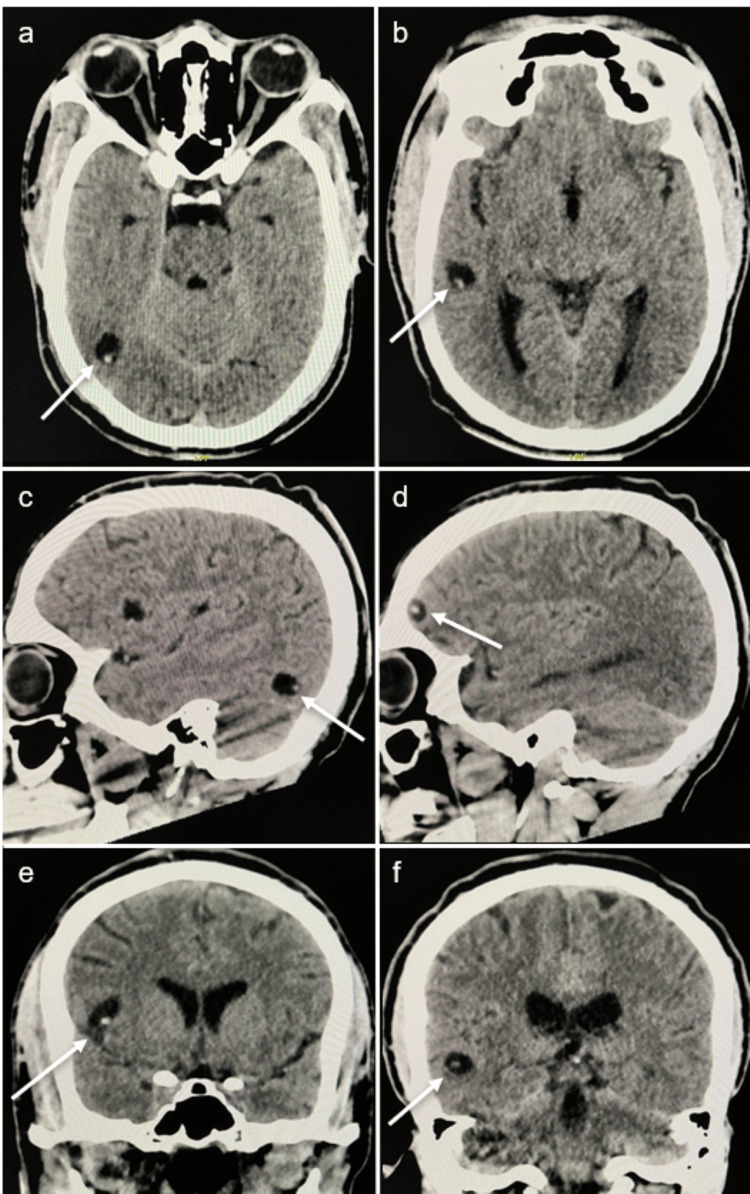
Nonenhanced computed tomography scan of the head showing four intraparenchymal cystic lesions with a small central hyperdense lesion representing scolex with minimal surrounded edema, along with multiple nodular calcifications that are highly suggestive of the vesicular, early colloidal, and calcified stages of intracranial NCC, as marked by the arrows, in the a-b) Axial, c-d) Sagittal, through left hemisphere, and e-f) Coronal views. NCC: neurocysticercosis

**Figure 2:**
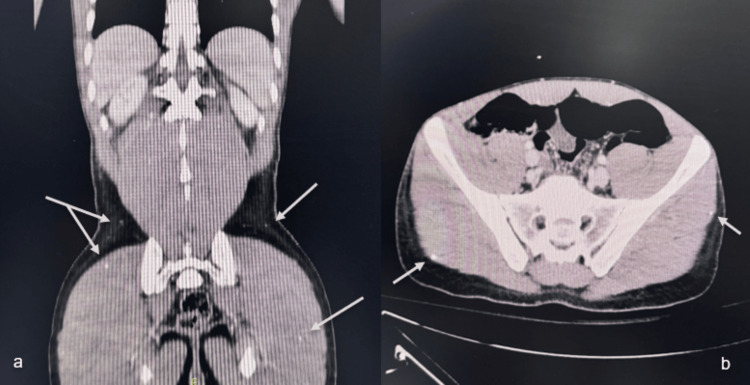
Enhanced computed tomography scan of the lower chest, abdomen, and pelvis revealing multiple muscular and cutaneous foci of calcification as marked by the arrows, in the a) Coronal, b) Axial views.

Computed tomography (CT) of the brain revealed four intraparenchymal cystic lesions with a small central hyperdense lesion representing a scolex that was surrounded by minimal edema, along with multiple nodular calcifications that were highly suggestive for vesicular, early colloidal, and calcified stages of intracranial NCC (Figures 1a-1f). Chest, abdomen, and pelvis CT revealed multiple muscular and cutaneous foci of calcification (Figures 2a-2b). Lumbar puncture was performed, and showed the following cerebrospinal fluid (CSF) results; CSF protein 0.29 g/L (Reference 0.15-0.4 g/L), CSF glucose 4.39 mmol/L (Reference 2.21-3.89 mmol/L), CSF total nucleated cell count 22 (Reference 0-5x10^6/L), CSF neutrophils 86 (Reference 0-6%), CSF lymphocytes 14 (Reference 40-80%), red blood cells 5578 (Reference 0-10x10^6/L), along with negative Gram staining, fungus, and acid-fast bacilli cultures. Electroencephalography performed while the patient was intubated and showed abnormal electrical activity consistent with epileptiform changes in the form of multiple sharp and spike waves, noted mostly in the temporal leads.

Further history was obtained from the patient following extubation and regaining complete consciousness. He denied any previous history of seizures, fever, night sweats, unintentional weight loss, visual disturbance, numbness, weakness, or head trauma. His social history included a habitual consumption of raw and undercooked meat, including pork, back home in South Africa.

Additional investigations were carried out, including stool analysis, which showed no ova or parasites, and serum cysticercosis IgG was tested using a cysticercosis IgG enzyme-linked immunosorbent assay, and the result was positive for this patient.

The patient was initially given a regimen of empirical ceftriaxone 2 gram once daily, linezolid 600 milligram twice daily, and acyclovir 600 milligram three times per day, along with dexamethasone at a dose of 4 milligram twice daily for two days for the presumptive diagnosis of meningoencephalitis before the lumbar puncture. A loading dose of levetiracetam 1000 milligrams was administered, followed by a maintenance dose of 500 milligrams twice daily. On the second day of admission, an ophthalmological evaluation confirmed no eye involvement or papilledema. The neurosurgery team recommended that no surgical intervention was needed. Therefore, the patient started on albendazole 400 milligrams twice daily, and ceftriaxone, linezolid, plus acyclovir were discontinued. Subsequently, he developed a hepatocellular pattern of deranged liver functions with an increase in alanine transaminase and aspartate transferase by five-fold from the upper limit of normal, while bilirubin and alkaline phosphatase remained within normal limits, with the absence of jaundice and coagulopathy. As a result, albendazole was discontinued. Ultimately, he was discharged on praziquantel 900 milligrams three times a day for a total of 14 days, in addition to oral prednisolone 60 milligrams with a tapering regimen by 10 milligrams every 5 days and levetiracetam 500 milligrams twice daily for seizure prevention.

**Figure 3:**
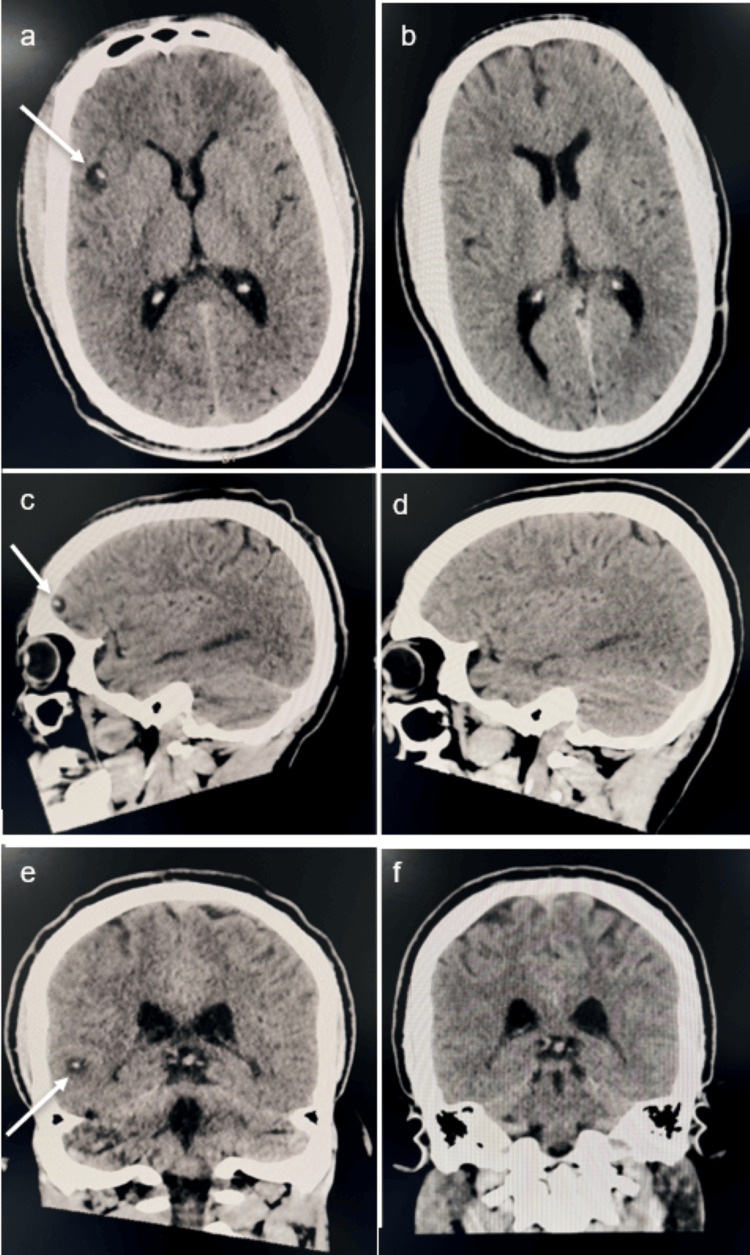
Nonenhanced computed tomography scan of the head showing significant regression of the multiple cystic lesions with a central scolex, as marked by the arrows, where a, c, e represent the lesions before anthelmintic drugs, and b, d, f show the regression of lesions after anthelmintic drugs, in the a-b) Axial, c-d) Sagittal, through left hemisphere, and e-f) Coronal views.

The patient was seen in the infectious disease and neurology clinics three weeks post-discharge. He was doing well, with no seizure recurrence or additional complaints. Liver enzymes returned to normal levels. A follow-up CT brain has been performed after four months of discharge and showed significant regression of the multiple cystic lesions with a central scolex (Figures 3a-3b).

## Discussion

Cases of NCC have been increasingly reported in the Arabian Peninsula [6]. Although a rare disease among nationals due to religious prohibition of pork ingestion, its incidence is growing, owing to the increase in immigrants [3,4]. The first case report described from Saudi Arabia was in 2003 by Al Shahrani et al., where they reported a young Saudi female patient who presented with focal seizures, initially suspected of having a tuberculoma. After magnetic resonance imaging of the brain revealed only a cystic lesion, and in light of negative serology testing for cysticercosis, she underwent surgical excision of the brain lesion. Biopsy revealed a degenerating larval cestode compatible with *T. solium*, confirming the diagnosis of neurocysticercosis [7]. Since then, eight cases have been reported from Saudi Arabia in the literature to date [3-7]. Most, except two [3,7], have been immigrants from Vietnam [3], Nepal [3], India [4,5], and Sudan [8]. Our patient had been an immigrant from South Africa. The majority of Saudi patients with NCC had acquired the infection through immigrant workers or household contacts [3,4]. Patients presented mainly with seizures and headaches, similar to our case, but some reported unusual psychiatric presentations with severe depression, suicide attempts, pseudobulbar manifestations, and dystonia [3,8]. An important differential diagnosis to consider, however, especially in endemic areas, is intracranial tuberculoma [4]. NCC is mainly diagnosed through its characteristic findings on neuroimaging studies based on cysticerci evolutionary stages that initially start with the vesicular stage, present as a cystic lesion with scolex, and then begin to degenerate. With an ongoing inflammatory reaction, the cyst will be surrounded by edema and ring enhancement, representing the colloidal stage. Then, the parasite will evolve further and shrink in size, with a thickened cystic wall representing a granular nodular stage; lastly, the cysticercus ends as calcified nodules [2,9]. Demonstration of a scolex within a cystic lesion on neuroimaging studies is one of the absolute criteria and is diagnostic of NCC [9]. This was present in our case; along with the resolution of cystic lesions upon follow-up imaging after treatment with albendazole and praziquantel, this provided robust support for the diagnosis of NCC [9].

Patients with viable parenchymal cysts are managed with a combination of anthelmintic drugs, corticosteroids to control the inflammation from degenerating cysts, and anti-seizure medications [1]. Albendazole is safe and effective in reducing the number of cysts as well as decreasing long-term seizure frequency. The combination of albendazole with praziquantel was associated with higher resolution of cysts, and thus is recommended for patients with more than two cysts [10]. Initially, due to the unavailability of praziquantel in our institution, the patient was started on albendazole alone; however, he couldn’t tolerate it due to hepatotoxicity and was switched to praziquantel. Cases described in Saudi Arabia have either used albendazole or praziquantel, according to availability [3-7]. There are no controlled trials comparing anti-seizure medications in the management of NCC-associated seizures; thus, the choice of agent should be guided by local availability/cost, potential side effects, and drug interactions [10]. We chose levetiracetam in our case due to its general tolerability and fewer drug-drug interactions.

## Conclusions

NCC, once considered rare in Saudi Arabia, has an increasing prevalence due to the increase in immigrant populations. The presentation mostly consists of seizures and headaches, and neuropsychiatric manifestations have been described. Physicians in Saudi Arabia should consider a diagnosis of NCC, especially in immigrants who present with new-onset seizures. We encourage the publication of such cases to determine the heterogeneity of presentations and increase the recognition of this disease in the country.
